# Acupuncture in Multidisciplinary Treatment for Post-COVID-19 Syndrome 

**DOI:** 10.1089/acu.2021.0086

**Published:** 2022-06-16

**Authors:** Robert J. Trager, Elise C. Brewka, Christine M. Kaiser, Andrew J. Patterson, Jeffery A. Dusek

**Affiliations:** ^1^ Connor Whole Health, University Hospitals Cleveland Medical Center, Cleveland, OH, USA.; ^2^ Department of Reproductive Endocrinology, School of Medicine, Case Western Reserve University, Cleveland, OH, USA.; ^3^ University Hospitals Harrington Heart and Vascular Institute, University Hospitals Cleveland Medical Center, Cleveland, OH, USA.; ^4^ Department of Family Medicine and Community Health, School of Medicine, Case Western Reserve University, Cleveland, OH, USA.

**Keywords:** acupuncture, COVID-19, complementary therapies, Traditional Chinese Medicine, pericardial effusion, exercise

## Abstract

**Background:**

Post-COVID syndrome (PCS) is a complex, multisystem illness that may follow SARS-CoV-2/COVID-19 infection. As there is limited evidence for individual therapies and no singular treatment for PCS, guidelines endorse a multidisciplinary approach. This is a case report of a patient with PCS benefiting from a comprehensive approach including acupuncture with symptom-titrated physical activity (STPA).

**Case:**

A 50-year-old woman presented from a Long-COVID Clinic referral to an outpatient hospital-affiliated acupuncturist. She had 8 months of fatigue, anosmia, chest pressure, palpitations, and other symptoms following mild assay-confirmed COVID-19. Prior/concurrent medical testing revealed multisystem-inflammatory involvement (pericardial effusion, thyroid dysfunction, and elevated d-dimers). Cardiology/pulmonology cleared the patient for exercise to tolerance considering that serious pathology was absent. The acupuncturist's Traditional Chinese Medicine impression was of Qi Deficiency of the Heart, Lung, Spleen, and Kidney. This patient received 7 sessions of scalp, auricular, and body acupuncture. Physical-therapist (PT)-led STPA began 1-week post-acupuncture, involving 6 30-minute exercise sessions while monitoring her heart rate, with as-needed rest.

**Results:**

The patient's chest pressure and palpitations resolved after 1 acupuncture treatment. With 6 additional treatments, spanning 9 weeks, overlapping with PT-led SPTA, she recovered completely and resumed her normal exercise.

**Conclusions:**

Acupuncture appeared to facilitate PCS recovery. However, the independent effects of acupuncture are less clear, given the concurrent STPA/exercise therapy, and should be explored using large study designs. Acupuncture is an attractive potential PCS therapy, considering its holistic approach and that it may be added to a multidisciplinary, guideline-concordant regimen.

## INTRODUCTION

Post-COVID syndrome (PCS), also called long-COVID syndrome/condition, is a complex, multisystem illness,^1^ diagnosed when symptoms persist 1–3 months post-COVID-19/SARS-CoV-2.^2^ Given that there is no singular PCS treatment, guidelines and expert panels recommend a multidisciplinary approach.^1–3^ PCS is common, with 37% of patients having at least 1 symptom 3 months' post-COVID.^4^ Potential PCS risk factors include female sex, worse initial COVID-19 severity, and more initial symptoms.^5^

The most-common PCS symptom is fatigue, affecting 58% of patients,^6^ followed by headache, attention disorder, hair loss, and dyspnea, and others, with life-threatening conditions being rare (e.g., stroke, myocarditis).^6^ Multiple-organ imaging abnormalities are found in 29% of patients who have PCS (i.e., in the lungs, heart, kidneys, liver, spleen, or pancreas).^7^

Evidence for PCS treatments is limited. A literature search of PubMed^®^ and Google Scholar (on November 1, 2021) yielded few cases of acupuncture used to treat PCS.^8,9^ Despite limited evidence, guidelines consider symptom-titrated physical exercise (STPA) to be an option for addressing PCS, with caution to not exacerbate symptoms.^10^

## CASE

### Patient Information

**FIG. 1.**
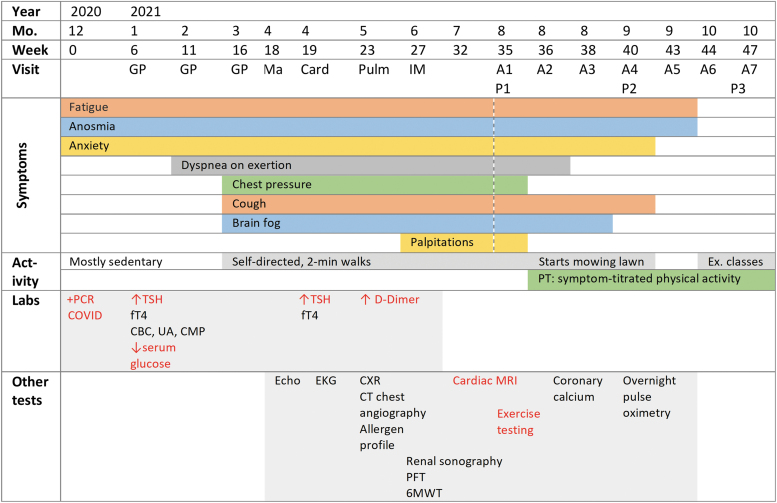
Clinical timeline, including year, month, and weeks post-COVID relative to first positive test: symptoms; activity levels; laboratory findings; and other tests. Red text indicates abnormal laboratory or other test findings. Dotted line indicates start of acupuncture. ↑ indicates elevated, ↓ indicates lowered, and + indicates positive. min, minute; Ex., exercise; PT, physical therapy; PCR, polymerase chain reaction; TSH, thyroid-stimulating hormone; fT4, free thyroxine; CBC, complete blood count; UA, urinalysis; CMP, comprehensive metabolic panel; Echo, echocardiogram; EKG, electrocardiogram; CXR, chest radiography; CT, computed tomography; PFT pulmonary-function testing; 6MWT, 6-minute walk test; MRI, magnetic resonance imaging.

A 50-year-old Caucasian woman (69 kg; 1.66 m, and body mass index 25), a never-smoker, nondiabetic, who consumed ≤5 alcoholic drinks per week presented via a Long-COVID Clinic referral to an integrative-medicine, outpatient clinic–based acupuncturist. The patient had fatigue, anosmia, ageusia, anxiety, dyspnea on exertion, chest pressure, dry cough, brain fog, and palpitations in August of 2021, 35 weeks post-SARS-CoV-2 positivity/COVID-19 illness (Fig. 1). She reported having fatigue for 1 month before this test; however, her initial SARS-CoV-2 nasal swab was negative (in November of 2020).

Post-COVID-19 to presentation, she became fatigued from performing everyday tasks (e.g., cooking). Pre-COVID-19, she had exercised regularly without experiencing any issue (performing Zumba^®^). Upon attempting returning to this exercise 16 weeks-post-COVID, she had to stop due to dyspnea, and reported that she “felt like [she] was going to have a heart attack.” Motivated to overcome fatigue, she would walk for 2-minute intervals to tolerance, later attempting brief periods of yard work.

Pre-COVID-19, the patient had long-standing anxiety that managed successfully with citalopram and buspirone; however, her anxiety became exacerbated post-COVID-19. Pre-COVID, she also had occasional temporal headaches and had undergone an appendectomy that was not recent. Her family history was significant for a cerebrovascular accident and aortic aneurysm (father), and arthritis and colonic polyps (mother). Pre-COVID-19 to current, she took an oral estrogen–progestin contraceptive, and an over-the-counter (OTC) multivitamin and (OTC) calcium/vitamin D.

This patient's COVID-19 course involved no acute/major complications, with a 2-week home-quarantine, and no hospitalization, no supplemental oxygen, and no antiviral/antibiotic treatments. She received a 2-dose COVID-19 mRNA vaccination (Moderna, Inc., USA) 6/10 weeks post-COVID-19, which did not change her PCS symptoms.

This patient provided written informed consent for publication of this case report.

### Ethics

This case report was deemed Not Human Subjects Research by the University Hospitals institutional review board. The authors obtained written consent from this patient for publication about her case.

### *Bio*medical Care 

The patient's primary care internal-medicine specialist evaluated her persistent COVID-19 symptoms (Fig. 1) An echocardiogram showed no acute findings; a normal ejection fraction (55%–60%); and a small, inconsequential patent foramen ovale. Her thyroid stimulating hormone (TSH) was serially elevated (6.55 and 4.10 mU/L) with reflex free thyroxine (fT4) borderline-low (1.00 and 0.96 ng/dL). Her complete blood count, urinalysis, and comprehensive metabolic panel were largely normal (mild hypoglycemia at 70 mg/dL). Her electrocardiogram, chest radiograph, and coronary calcium scan results were normal.

Primary care referred her to a cardiologist, whose examination revealed no abnormalities. Her blood pressure was 121/73 with a resting heart rate (HR) of 58 beats-per-minute (bpm). Her brain natriuretic peptide (46 pg/mL) and troponin-I (< 0.02 ng/mL) levels were normal and the results of a lipid panel were slightly abnormal (total cholesterol: 224 mg/dL). Her d-dimers were elevated (815 ng/mL; normal: ≤500). The cardiologist recommended self-directed-STPA with as-needed rest.

**FIG. 2.**
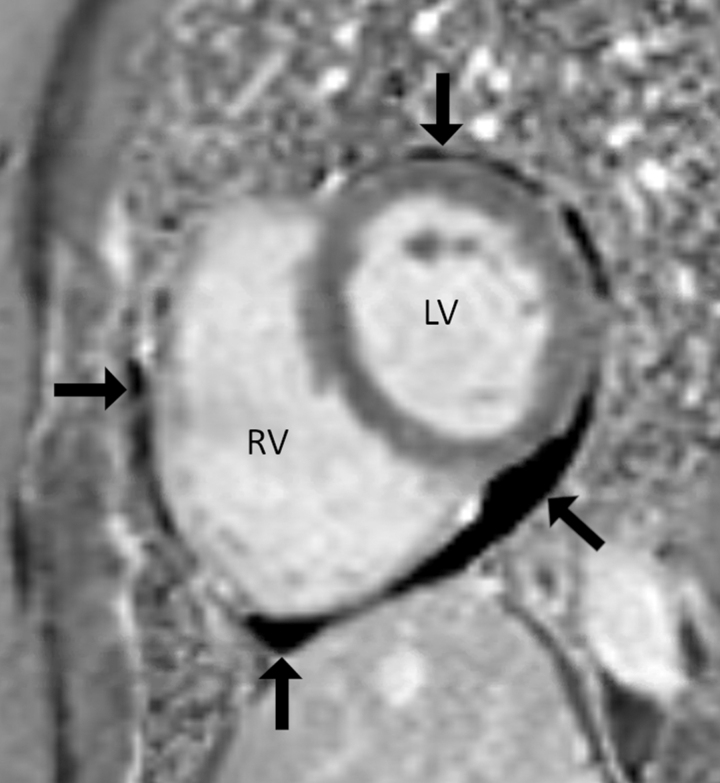
Pericardial effusion (*arrows*). Cardiac magnetic resonance image, short-axis view, and phase-sensitive inversion recovery sequence. *Arrows* indicate a hypointense signal extending circumferentially around the heart, which represents a pericardial effusion. There is no pericardial thickening in this sequence, or enhancement with gadolinium in the other sequences, which are not shown. LV, left ventricle; RV right ventricle.

Primary care later consulted a pulmonologist (via the hospital's Long-COVID Clinic), who evaluated for myocarditis, pulmonary embolism, and reactive airway disease. Her respiratory allergen profiles (ImmunoCAP™ and immunoglobulin E), pulmonary-function tests, and computed tomography chest angiography results were normal. Cardiac magnetic resonance imaging (MRI) revealed normal biventricular size/function without gadolinium enhancement to suggest a prior infarct or infiltrative process. There was no aberration in T1/T2-weighted images to suggest underlying inflammation. A nonspecific, small, circumferential pericardial effusion was seen, without pericardial hyperenhancement (Fig. 2). A 6-minute walk test (6MWT) provoked 4/10 dyspnea and 7/10 fatigue over normal-for-age distance. Her overnight pulse oximetry (Virtuox, Inc.) revealed no nocturnal desaturation, indicating no need for supplemental oxygen. The pulmonologist referred her for a physical therapist (PT)–led SPTA exercise program.

### Acupuncture

The initial Traditional Chinese Medicine (TCM) impression, based on patient symptoms and tongue/pulse examination (Table 1) was of Qi deficiency of the Heart, Lung, Spleen, and Kidney. The goal was to move Qi and tonify these organs, and weekly treatments were recommended. The patient was treated by 2 TCM–licensed acupuncturists (LAcs), with the second acupuncturing treating the patient during visits 2–7. Acupuncture needles (Seirin,^®^ Weymouth, MA, USA) were 0.20 × 30 mm (for meridian acupoints), 0.16 × 15 mm (for ear points), and 0.18 × 30 mm (for scalp points). Auricular needles were inserted before body needles, with insertion depths varying from 0.1 to 1.0 *cun*. Thirty-minute needle retention, with De Qi and twisting and lifting techniques were well-tolerated.

**Table 1.** Acupuncture Diagnosis, Examination Findings, and Treatments

Visit	1	2	3	4	5	6	7
Diagnosis	NR	LU Qi *xu* & HT/SP Qi *xu*	LU Qi *xu*, HT/SP Qi *xu*, & KI Yang & Yin *xu*	LR Yang rising, LU Qi *xu*, SP Qi *xu*, & KI Yang and Yin *xu*	LR Yang rising, LU Qi *xu*, SP Qi *xu*, & KI Yang & Yin *xu*	LR Yang rising, LU Qi *xu*, & SP Qi *xu*	LR Yang rising, & LU Qi xu
Tongue	NR	Thin white coating, red pink body & dry	Thin white coating, red pink body & dry	Sticky white coating, dusky pink red body & dry	Thin white coating, dusky red, dry	Thin white coating, dusky red pink, moist & prickles	Thick white coating, dusky pink body, dry & no sublingual veins visible
Pulse	NR	(R) Wiry (L) Floating	(R) Soft (L) Taut deep weak Qi	Moderate	(R) Soggy (L) Thin wiry	(R) Soggy (L) Thin deep	(R) Floating (L) Deep wiry, thin deep Qi
Acupoints	GV 20 (B) PC 6, ST 36, SP 6 & LR 3 *Scalp:* Head & (B) Thoracic *Auricular:* (L) NADA	GV 20, (B) KI 3, ST 36, LU7, PC6 (R) LI 4, GB 41, (L) LR 3, TE 5 & SP 6 *Auricular:* (R) NADA	GV 20, (B) KI 3, ST 36, LU 7, SP 6, SP 7, SP 9, ST 25, (R) GB 41, PC 6 & (L) TE 5	GV 20, GV 24 (B) SP 10, SP 9, SP 6, KI 3, ST 36, LU 7, (R) GB 41, LI 5, LI 11, (L) TE 5, LR 3 & LI 10	GV 20, (B) GB 8, LR 3, ST 36, PC 6 (R) SP 9, LI 4 (L) TE 5, LI 11 & GB 34	GV 20, (B) LR 3, SP 6, SP 7, SP 9, ST 36, LI 4, PC 6, LU 7 & (L) LI 11	GV 20, (B) LR 3, GB 8, GB 34, GB 41, LI 4, GB 20, (L) PC 6 & (R) LU 7
Auricular beads	—	—	(B) *Shenmen* & *SanJiao*	(B) *Shenmen*, MidBrain, Lung, & Adrenal		(B) *Shenmen* & Hungry point	(B) *Shenmen* & Hungry point

*xu* denotes Deficiency.

*Body points are:* GB, Gallbladder; GV, Governor Vessel (*Dumai*); HT, Heart; KI, Kidney; LI, Large Intestine; LR, Liver; LU, Lung; PC, Pericardium; SI, Small Intestine; SP, Spleen; ST, Stomach; & TE, Triple Energizer.

The 5 auricular NADA points are *Shenmen*, Sympathetic, Liver, Kidney, and Lung.

NR, not recorded; (B), bilateral; NADA, National Acupuncture Detoxification Association; (R), right; (L), left.

Stomach (ST) 36 and Large Intestine (LI) 4 acupoints were needled in combination to clear the body of lingering Heat, as seen by a red-pink tongue, and Dampness, as observed by a soft pulse (Table 1). These points were also selected along with Lung (LU) 7 to fortify the patient against her Lung and Spleen Deficiencies. Triple Energizer (TE) 5 and Gallbladder (GB) 41 acupoints were needled in combination for strengthening her *Wei* Qi (immunity), draining her Dampness, and regulating her Ya*ngweimai* and *Daimai*. These points were effectively selected to harmonize defensive and nutrient Qi, move Yin through the channels, smooth Liver Qi, and regulate the *Shao Yang*.

### Physical Therapy

Exercise testing revealed greater-than-expected HR increase during 1-floor stair-climbing (95 bpm, Polar-H10 Sensor), with a mild, transient decrease in SpO_2_ (96%). STPA was delegated to a PT-assistant, targeting a HR 119–136 bpm (70%–80% maximum for patient's age) for 30–40 minutes with as-needed rest. Intensity-per-session increased dependent on patient tolerance and HR stability (See Table 2). 

**Table 2.** Symptom-Titrated Physical Activity Program Supervised via Physical Therapy

*Weeks post-COVID*	*36*	*37*	*38*	*39*	*40*	*46*
*Visit*	*1*	*2*	*3*	*4*	*5*	*6*
NuStep^®^ cross-trainer (minutes)	7	10		10	10	10
Mini squats (reps)	60		60			
Standing hip abduction (reps per side)	15	15	15			
6″ step-ups (reps per side)	60		60			
Standing hip extension (reps)	15	15	15			
Sets of stairs (reps)	3	3	3	6	6	6
Biceps curls (3 lbs, reps)	5	15	15			
Deltoid front raise (2 lbs, reps)	15		15			
Deltoid lateral raise (2 lbs, reps)	15		15			
Deltoid raises (reps)						
Forward lunges (reps)		60		60	60	60
Carioca steps (reps)		60				
Wall push-ups (reps)		30				
Squat with military press (3 lbs, reps)		60				
Side steps (yellow Thera-Band,^®^^a^ reps)				60	60	60
Monster walk (yellow Thera-Band, reps)				60	60	60
Mini squats with military press (5 lbs, reps)				60	60	60
Lateral lunges with weights (2 lbs, reps)				60	60	60
Hip raise (reps)				60	60	60

Note that the first physical therapy appointment at 35 weeks' post-COVID-19 is not shown, as this was an evaluation with no exercise therapy performed.

^a^ A yellow Thera-Band^®^ provides 1–6 lbs of resistance.

lbs, pounds; reps, repetitions.

## RESULTS

This patient reported feeling relaxed immediately after her first acupuncture treatment, and noticed an absence of chest pressure and palpitations later. At acupuncture visit 2, which began a time of overlap her with PT-led-STPA, she reported having more energy. At visit 3, she reported being able to partially mow her lawn without dyspnea, and she also noted olfactory improvement. At visit 4, she noted further improvements in energy and reduced coughing. At visit 5, she reported feeling “awake again,” without brain fog. She did her first heavy yardwork since pre-COVID-19. At visit 6, she noted improved energy and had resumed her usual exercise classes. Progress was maintained at visit 7, in which she was treated for a typical/pre-COVID-19 temporal headache. The patient was doing well without a PCS relapse 1-month later.

## DISCUSSION

To the current authors' knowledge, this case is the first to highlight potential benefits of acupuncture within multidisciplinary PCS-treatment framework. Limitations included a lack of standardized patient-reported outcome assessments. Repeat testing (e.g., MRI, d-dimers) could have evaluated sequential changes. Recovery could be explained by the natural history of PCS. Findings from this case may not be broadly generalizable.

This patient had multisystem involvement affecting the heart (pericardial effusion) and thyroid (TSH), while her elevated d-dimers suggested systemic inflammation and hypercoagulability.^5^ These findings are relatively common in PCS, with elevated d-dimers in 6%–39% of cases,^6^ pericardial effusion in 27% of cases, and hypothyroidism in 5%–12% of cases.^11,12^ Although a pre-COVID baseline was unavailable, and confirmatory thyroid testing (e.g., fT3, antithyroid antibodies) was not conducted, PCS-related hypothyroidism/thyroiditis could explain her fatigue and anosmia.^13^ PCS-related pericardial effusion alone was unlikely to explain her chest pain, dyspnea, and palpitations.^14^

According to a theory from Wu You-ke (ce 1582–1652), illnesses may persist when pathogenic Qi becomes lodged in the *moyuan* (membrane source)—an energetic-anatomical area between the heart and diaphragm—and is transmitted through the Triple Energizer.^15^ Some TCM researchers have viewed COVID-19 as a pathogen affecting the *moyuan*, and multiple *Zang* organs (Lung, Heart, Kidney, Liver, Spleen) via its association with the Triple Energizer.^16,17^ This concept could apply to the current case, given the heart/lung symptoms, pericardial effusion, chronicity, and acupuncture pulse/tongue diagnosis of Qi Deficiency.

Acupuncture is an intriguing prospective therapy for PCS given acupuncture's holistic, individualized approach, which parallels the varying symptoms and organ involvements of PCS. However, there may be some commonalities in TCM approaches to COVID/PCS, as 2 acupoints used in this case are among those most commonly used in acute COVID-19 (LI 4/ST 36).^18^ Generally, stimulation of ST 36 has been found to increase deep circulation,^19^ possibly through nitric oxide production.^20^ Furthermore, 1 article proposed that acupuncture's positive circulatory effects could benefit patients who have COVID with elevated d-dimers.^21^

One notable finding in this case was that the patient's chest pressure/palpitations were alleviated rapidly when she started receiving acupuncture, preceding her PT-led-STPA. The current authors suspect that this response was related to the autonomic effects of acupuncture, rather than a rapid reduction in her pericardial effusion. Specifically, the rapid resolution of her chest pressure and palpitations could be explained by acupuncture upregulating parasympathetic activity^22^ and producing an anxiolytic effect.^23^

The effects of acupuncture are intermingled with those of STPA in this case. However, the patient had already been exercising to tolerance preceding her PT-led-exercise without changes in PCS symptoms. Rapid improvements after starting acupuncture were suggestive of an added benefit of this therapy. Conversely, formal STPA supervision plausibly provided the patient with a beneficial environment to exercise safely to her HR capacity and progress in recovery. The current authors suspect that the acupuncture and exercise were synergistic, with PT-led-STPA helping her regain strength/endurance after months of reduced activity, and acupuncture conceivably facilitating abrupt reduction of her PCS symptoms.

This case highlights the potential utility of acupuncture within a multidisciplinary approach to a patient who had PCS without any serious underlying medical comorbidity. Initiation of acupuncture, coupled with STPA, coincided with surpassing a plateau in her PCS recovery. Studies examining the effects of acupuncture on PCS could incorporate low-cost, practical measures of recovery, such as the 6MWT of HR and SpO_2_, which were valuable in the current case.

## CONCLUSIONS

Acupuncture may be part of a multidisciplinary approach for PCS treatment pending appropriate clinical trials. In this case report, it appears that the patient benefited from the acupuncture but can not be extrapolated to other cases dealing with PCS until further statistical data is obtained from future studies.

### The Patient's Perspective

The patient described her experience with her treatment as follows:

My post-COVID experience was frustrating until multiple medical tests cleared me to focus on recovery, which included acupuncture and physical therapy. I had never tried acupuncture but was open to any solution that might make me feel better. I was surprised at the immediate impact I felt after my first acupuncture treatment and how quickly I started to return to normal life. Acupuncture not only helped me feel better physically, it gave me hope I would finally recover from long-COVID.

## AUTHORs' CONTRIBUTIONS

Drs. Trager, Kaiser, Dusek and Mrs. Brewka conceived of the case report. Dr. Patterson interpreted imaging findings. All of the authors drafted, provided intellectual content, critically revised, and approved the final article.
